# Case Report: Incidentally Discovered a Rare Cystic Lesion of Liver: Multicystic Biliary Hamartoma

**DOI:** 10.3389/pore.2021.628323

**Published:** 2021-03-30

**Authors:** Wentao Mu, Peng Su, Shanglei Ning

**Affiliations:** ^1^ Department of Hepatobiliary Surgery, General Surgery, Qilu Hospital of Shandong University, Ji’nan, China; ^2^ Department of Pathology, Qilu Hospital of Shandong University, Ji’nan, China

**Keywords:** multicystic biliary hamartoma, cystic lesion of liver, laparoscopic resection, liver, immunohistochemistry

## Abstract

Multicystic biliary hamartoma (MCBH) is an extremely rare cystic lesion of the liver. A 37-year old male patient was admitted to our hospital for incidentally discovered hepatic cystic lesions on abdominal ultrasonography. Abdominal contrast-enhanced computed tomography (CT) showed a multilocular cystic lesion in the segment VI, with mild enhancement in the septae and peripheral wall within the lesion. Only alanine transaminase (ALT) and carbohydrate antigen 19–9 (CA19–9) increased slightly above normal value. Preoperative tests suggested possibility of a benign mucinous cystic neoplasm (MCN) or intraductal papillary neoplasm of the bile duct (IPNB). Laparoscopic complete resection of the lesion was performed. Histopathological examination showed numerous variably sized ductal structures surrounded by periductal glands and fibrous connective tissues containing small blood vessels and smooth muscle bundles. Immunohistochemical staining (IHC) revealed that dilated ducts were positive for cytokeratin CK19, characteristic for biliary tract. Histopathological findings confirmed diagnosis of multicystic biliary hamartoma (MCBH). No recurrence occurred during 6 months follow-up. In conclusion, MCBH should be differentiating from hepatic cystic lesion and could be resected laparoscopically safely.

## Introduction

Multicystic biliary hamartoma (MCBH) is a very rare cystic lesion of the liver and its pathological features were first described in 2005. Although clinical course of MCBH is relatively benign, because of its rarity, the natural history and appropriate treatment are not well established. It is difficult to identify MCBH preoperatively. In this report we present the first case of MCBH fully resected by laparoscopic surgery.

## Case Presentation

**FIGURE 1:**
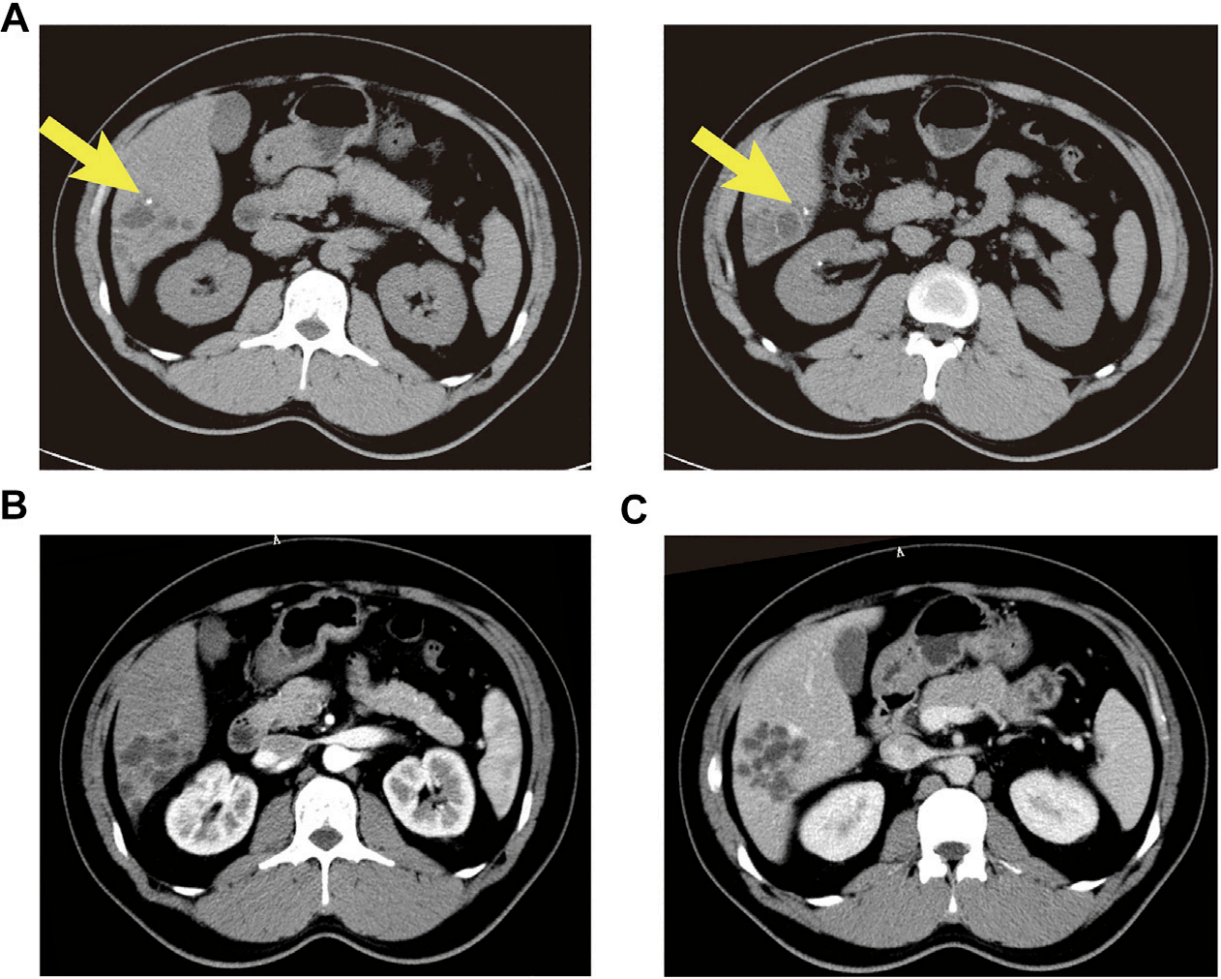
Abdominal contrast enhanced CT showed an intrahepatic multicystic mass in the segment VI of the liver. **(A)** Calcification was observed in dilated ducts (plain phase arrow); **(B)** The enhancement of the septa and peripheral wall within the cystic lesion (arterial phase); **(C)** The lesion showed a low-density honeycomb-like appearance (venous phase).

**FIGURE 2:**
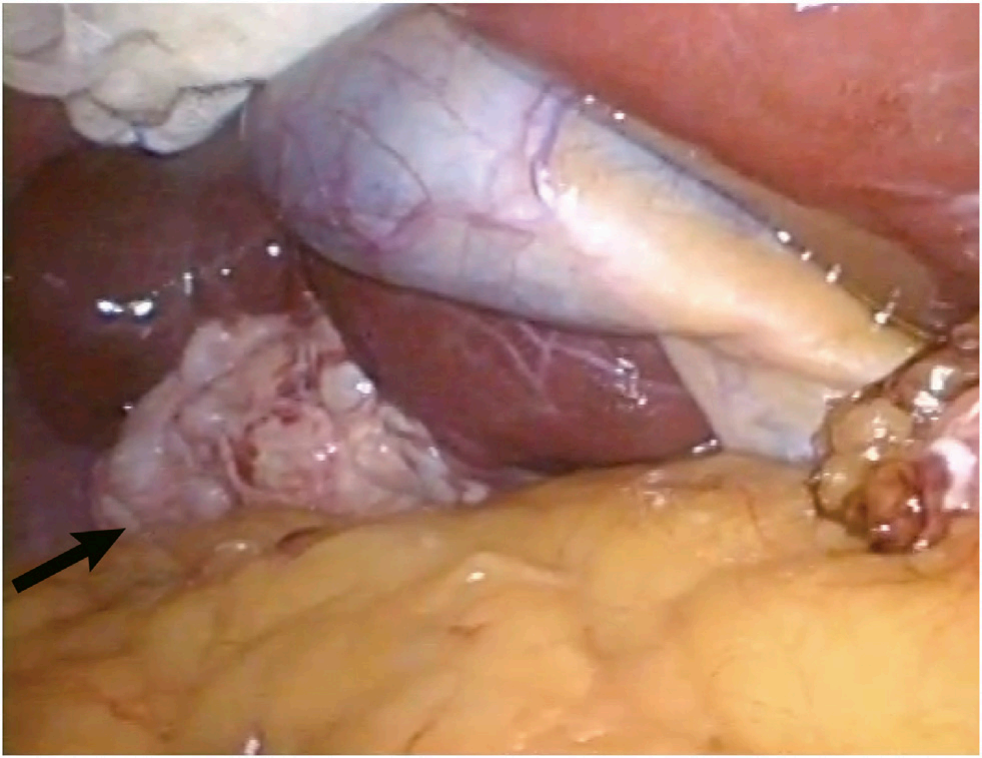
The grey-white and typical multicystic appearance of the lesion was shown during laparoscopic operation (arrow).

**FIGURE 3:**
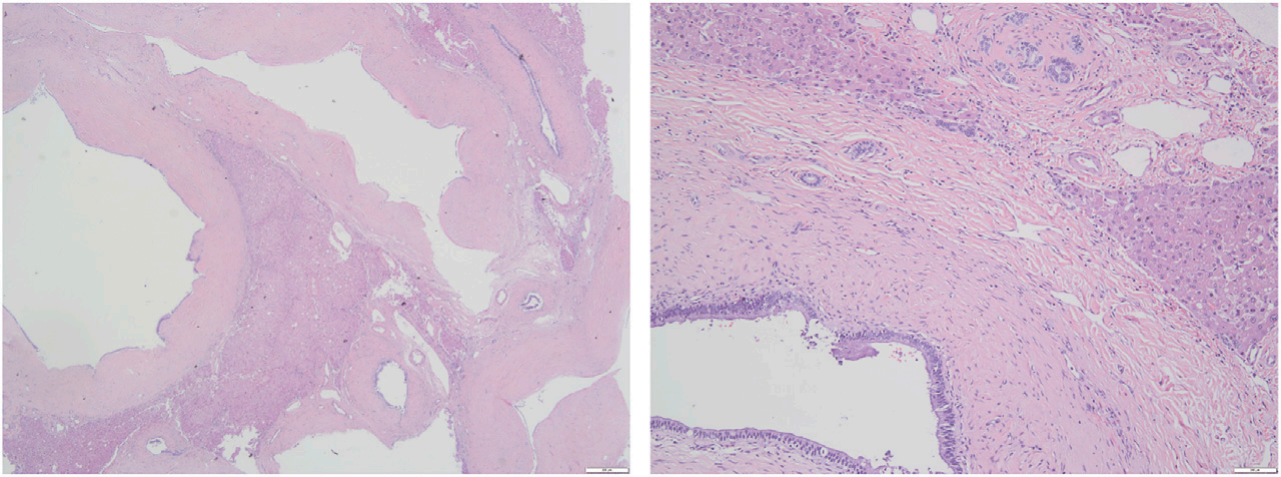
Histological findings of the resected lesion tumor (Hematoxylin and eosin staining). **(A)** The lesion consisted of dilated cystic ducts surrounded by the connective tissue of smooth muscle and capillaries (×20); **(B)** Ducts are lined by columnar epithelium (×100).

A 37-year old male patient was found to have a multicystic mass with the size 7.7*5.6 cm in the right hepatic lobe incidentally on abdominal ultrasonography. Then he was admitted to our hospital for mild abdominal distention after eating. Neither other significant clinical symptoms nor positive signs were found after further physical examination. Further examinations including liver function、tumor markers and abdominal contrast-enhanced computed tomography (CT) scan were performed. The laboratory results were as follows: alanine transaminase (ALT), 67U/L (normal: 9–40 U/L); carbohydrate antigen 19–9 (CA19–9), 65 U/mL (normal: 0–39 U/mL); other liver function and tumor markers were all in normal range. Abdominal CT revealed a multilocular cystic lesion in the segment VI of liver (Figure 1C). Interestingly, small calcification was found on precontrast CT (Figure 1A) and the septa and peripheral wall within the cystic lesion showed mild enhancement on postcontrast (Figure 1B). Based on these imaging results, we suspected that the lesion could be mucinous cystic neoplasm (MCN) or intraductal papillary neoplasm of the bile duct (IPNB) of the liver before operation. Laparoscopic complete resection of the lesion was performed (Figure 2). Intraoperative findings showed a lesion was grey-white lesion with multicystic surface. No enlarged lymph nodes were found during the operation. The cut surface of the resected specimen has many dilated ductal structures and hepatic parenchyma between these ducts, while bile-like material was observed within some ducts. Intraoperative frozen histopathological examination suggested preliminary diagnosis of biliary cystadenoma. Histopathological findings of formalin fixed paraffin embedded tissue on revealed periductal glands surrounded by connective tissue containing smooth muscle and small blood vessels but no ovarian-like stroma (Figure 3). Immunohistochemistry showed CK19 positivity of the dilated ducts epithelium and the small vessels were positive for CD30. Final histopathological diagnosis confirmed a multicystic biliary hamartoma (MCBH). After the surgery serum CA19–9 level dropped to normal range (28 U/mL) and there were no signs of recurrence 6 months after surgery.

## Discussion

Multicystic biliary hamartoma (MCBH) is a very rare cystic lesion of the liver, which was first reported in 2005 as solitary bile hamartoma (Kobayashi et al., 2005). The pathogenesis of MCBH is still not entirely understood and Zen et al suspected it may be related to abnormal development of biliary ducts or embryonal foregut (Zen et al., 2006). There are only 15 cases described in the literature to date (Table1). Mean age of the 16 patients (including the presented case) is 54 years, and the gender distribution is ten male and six female. Most of the lesions were located in segment III (six patients) and VI (six patients), and the left lobe lesions located in segment IV and VII. Two patients had hepatitis and increased CA19–9 serum level was observed in three patients. Eleven MCBHs were found near to the liver surface. Pathological results found small stones in dilated ducts in our case, probably it’s the reason for minor elevation of serum CA19–9 level. Morinaga et al reported a 53-year-old MCBH patient with extremely elevated CA19–9 but without evidence of cholangitis (Morinaga et al., 2017). Although the reason for the increase of CA19–9 level in our case is not definite, it decreased to the normal range after resection in both cases.

**TABLE 1:** Multicystic biliary hamartoma cases reported to date.

Case no.	Authors	Publication year	Age (years)	Gender	Size (cm)	Location/near liver surface	Surgical treatment	Co-existing disease
1	Kobayashi et al. (2005)	2005	30	M	3.6	Seg. VI/yes	Partial resection	None
2	Zen et al. (2006)	2006	59	M	4.2	Seg. IV/yes	Left hepatectomy	None
3			70	F	1.8	Seg. III/yes	Segmentectomy	Hepatitis C cirrhosis
4			69	F	2.8	Seg. III/no	Segmentectomy	None
5	Kai et al. (2008)	2008	55	M	5.0	Seg. VI/no	Partial resection	Hepatitis B
6	Ryu et al. (2010)	2010	45	M	2.0–3.5^a^	Seg. VII/no	Partial resection	None
7			58	M	-	Seg. III/no	Partial resection	None
8			55	F	-	Seg. VI,VII/no	Partial resection	None
9	Song et al. (2013)	2012	52	M	2.7	Seg. III/yes	Partial resection	None
10	Beard et al. (2014)	2014	48	F	4.7	Seg. VII/yes	Extended right hepatectomy	Hepatitis C
11	Yoh et al. (2014)	2014	69	M	3.0	Seg. III/yes	Left hepatectomy	None
12	Fernández-Carrión et al. (2014)	2014	60	F	5.0	Seg. VI/yes	Partial resection	CA 199 slightly elevated
13	Tominaga et al. (2015)	2015	26	M	10.0	Seg. V,VI/yes	Right hepatectomy	None
14	Morinaga et al. (2017)	2017	53	M	12.0	left lobe of the liver/yes	Left hepatectomy	Ca 199 extremely elevated
15	Ogura et al. (2018)	2018	77	F	12.0	Seg. III/yes	Partial resection	None
Present case		2020	37	M	8	Seg. VI/yes	Laparoscopic partial resection	CA199 slightly elevated

^a^ Ryu et al. actually reported imaging manifestations of four cases, but one of them was a 70-year-old female, which was reported in a 2006 paper by Zen et al. (2006). This table only summarizes the other three cases. This paper provides a range of tumor sizes, but does not provide specific measurements for each tumor.

Histologically, MCBH consisted mainly of several ductal structures of different size surrounded with periductal glands and fibrous connective tissues containing small blood vessels and smooth muscle bundles. The dilated ducts contain bile-like material and are positive for biliary-type cytokeratin CK19 (Zen et al., 2006). In 2010, Ryu et al first summarized the imagining features of MCBH: 1) the cut-surface of MCBH showed honeycomb-like appearance and cysts are relatively even in size (above 1 cm); 2) the cystic wall and/or septa within the lesion showed mild enhancement; 3) there was normal hepatic parenchyma within the coalescent lesions; 4) the lesion generally located near the liver surface and protruded from the liver (Ryu et al., 2010). Other reports showed that MCBH also could occur deeper within the hepatic parenchyma (Kai et al., 2008). Cystic liver lesion is the imaging characteristic of many diseases including von Meyenburg Complex (VMC), mesenchymal hamartoma, MCN, Caroli’s disease, and IPNB (Pitchaimuthu and Duxbury, 2017). Because of the overlapping imaging appearances MCBH should also be considered for differential diagnosis of cystic lesion in liver.

Von Meyenburg Complex (VMC) is also known as biliary microhamartoma was firstly reported in 1918 (Shi et al., 2015). Histological findings of VMC also showed irregular or round dilated ductal structures lined with columnar or cuboidal epithelium surrounded by abundant fibrous stroma. Contrary to the relatively larger size of nodules in MCBH (1–5 cm), the size of nodules in biliary microhamartomas are usually between 2 and 5 mm in diameter. Though VMC is generally considered innocuous, in exceptional cases association with cholangiocarcinoma or hepatocellular carcinoma have been described (Sugawara et al., 2018; Heinke et al., 2008). MCBH is reported to be a benign disease and no recurrence or distant metastases have been reported in the literature (Tominaga et al., 2015). Liver mesenchymal hamartoma is characterized by loose mesenchyme or distorted slit like ductal structures and hepatocyte cords, which were not observed in our case (Stocker and Ishak, 1983).

Mucinous cystic neoplasm (MCN) is another relatively common disease which should consider in the differential diagnosis. It is a true neoplasm with malignant potential. The cystic spaces are lined by mucin-producing epithelium. MCN is defined as a multilocular liver lesion and the pathological characteristic is usually with an “ovarian-like” stroma, not like periductal glands and abundant fibrous stroma between dilated ducts as it is in MCBH (Song et al., 2013).

IPNB is characterized by biliary cystic lesion or dilated bile ducts with or without mucin production similar with intraductal papillary mucinous neoplasm (IPMN) in pancreas. Papillary components or adenocarcinomas were prevalent in such cystic lesion or dilated ducts, usually resulting biliary obstruction or liver function damage; however, solid process was completely absent in the cystic wall in MCBH. Furthermore, MRCP showed that the lesion always communicated with main hepatic ducts, which is different from MCBH (Tominaga et al., 2015).

Caroli’s disease is a type of congenital disease with dilation of intrahepatic ducts, which was first reported in 1958 (Caroli et al., 1958). With the progression of the disease, chologenic infection and malignant transformation are the main serious complications. Cystic dilated structures in Caroli’s disease arise from existing bile ducts therefore Caroli’s disease ducts always communicate with bile duct system, while the MCBH derives from hamartomas or abnormal ducts and do not communicated with hepatic ducts (Ryu et al., 2010).

In conclusion, we reported the first laparoscopic resection of MCBH with minor CA19–9 elevation. MCBH should be taken into consideration in differential diagnosis of solitary cystic lesions in liver.

## Data Availability

The original contributions presented in the study are included in the article/Supplementary Material, further inquiries can be directed to the corresponding author.
